# Improving health worker performance of abortion services: an assessment of post-training support to providers in India, Nepal and Nigeria

**DOI:** 10.1186/s12978-017-0416-0

**Published:** 2017-11-21

**Authors:** Janie Benson, Joan Healy, Sally Dijkerman, Kathryn Andersen

**Affiliations:** Ipas, P.O. Box 9990, Chapel Hill, NC 27514 USA

**Keywords:** Abortion care, Health worker performance, Performance measures, Quality of abortion care, Post-training provider support, Induced abortion, Postabortion care (PAC), Postabortion contraception

## Abstract

**Background:**

Health worker performance has been the focus of numerous interventions and evaluation studies in low- and middle-income countries. Few have examined changes in individual provider performance with an intervention encompassing post-training support contacts to improve their clinical practice and resolve programmatic problems. This paper reports the results of an intervention with 3471 abortion providers in India, Nepal and Nigeria.

**Methods:**

Following abortion care training, providers received in-person visits and virtual contacts by a clinical and programmatic support team for a 12-month period, designed to address their individual practice issues. The intervention also included technical assistance to and upgrades in facilities where the providers worked. Quantitative measures to assess provider performance were established, including: 1) Increase in service provision; 2) Consistent service provision; 3) Provision of high quality of care through use of World Health Organization-recommended uterine evacuation technologies, management of pain and provision of post-abortion contraception; and 4) Post-abortion contraception method mix. Descriptive univariate analysis was conducted, followed by examination of the bivariate relationships between all independent variables and the four dependent performance outcome variables by calculating unadjusted odds ratios, by country and overall. Finally, multivariate logistic regression was performed for each outcome.

**Results:**

Providers received an average of 5.7 contacts. Sixty-two percent and 46% of providers met measures for consistent service provision and quality of care, respectively. Fewer providers achieved an increased number of services (24%). Forty-six percent provided an appropriate postabortion contraceptive mix to clients. Most providers met the quality components for use of WHO-recommended abortion methods and provision of pain management. Factors significantly associated with achievement of all measures were providers working in sites offering community outreach and those trained in intervention year two. The number of in-person contacts was significantly associated with achievement of three of four measures.

**Conclusion:**

Post-training support holds promise for strengthening health worker performance. Further research is needed to compare this intervention with other approaches and assess how post-training contacts could be incorporated into current health system supervision.

## Plain English summary

Many evaluations and interventions have focused on health worker performance in low- and middle-income countries. Few have described how support following clinical training, including clinical and problem-solving support, improves heath workers’ clinical practice. This paper reports the results of an intervention with 3471 abortion providers in India, Nepal and Nigeria. Following abortion care training, providers received in-person visits and phone and/or email contacts by a clinical and programmatic support team for one year. This support was designed to address their individual practice issues. Measures were established to assess providers’ performance.

Providers received an average of six contacts. Sixty-two percent of providers consistently provided abortion services. Fewer providers increased the number of services provided (24%). Forty-six percent provided an appropriate mix of postabortion contraceptives to clients. Most health workers provided high quality services, including use of abortion technologies recommended by the World Health Organization and pain management. Working in sites offering community outreach was associated with achievement of all performance measures. The number of in-person contacts was associated with achievement of three of four measures.

Health worker support following training holds promise for strengthening provider performance. Further research is needed to compare this intervention with other approaches and assess how in-person contacts could be incorporated into current health system supervision.

## Background

Ensuring high-quality health worker performance is an ongoing challenge in low- and middle-income countries (LMICs) [1–3]. The needs of growing populations, inadequately resourced public health systems, and persistent shortages of skilled workforces all contribute to deficits in health worker practices [4]. Interventions have been implemented to improve health workers’ knowledge, attitudes, practices, job satisfaction and motivation, and in some instances, to enhance clinical and patient outcomes [2, 3].

Assessments of maternal-child health and family planning interventions are particularly common, especially in primary health care (PHC) settings [5–11]. These include various approaches to health worker supervision [6, 8–11]; mentoring, task-sharing and community outreach [12]; audit and feedback [2]; standards-based management and recognition of achievements [9]; provider training/refresher updates and follow-up outreach visits [13, 14]; and improvement collaboratives [15]. A few interventions demonstrated large effects in achieving desired outcomes, many had small to moderate effects, and others were inconclusive or had little or none.

A 2011 Cochrane Review of the effects of supervision on the quality of primary health care in LMICs concluded that supervision overall had uncertain effects although a separate review found that a number of individual studies documented beneficial results of supervision on health worker performance and PHC quality [8, 11]. Multi-faceted interventions appear to result in larger effects than single interventions [2, 3, 16], although a separate review notes a lack of clarity about the effectiveness of multi- versus single-component interventions [7].

An estimated 50 million induced abortions occur annually in developing countries, many taking place outside health facilities [17]. A large number, however, are carried out in health facilities in public, private and non-governmental organizational (NGO) sectors, and an estimated seven million women are treated for complications from unsafely-performed abortions each year [18]. These figures underscore the critical need to ensure that women are readily able to access facility-based care and that abortion providers offer high-quality services in those sites.

Results from a number of evaluation studies on interventions to improve abortion care (induced abortion and treatment of abortion complications/postabortion care [PAC]) in health facilities have been published. Most involved combinations of health worker training/refresher training on clinical services and counseling techniques, physical upgrades in sites, commodities support, on-site monitoring and technical assistance and educational outreach to the community. Outcomes included use of World Health Organization (WHO)-recommended technologies for abortion procedures, postabortion contraceptive uptake, client satisfaction with care, provider knowledge and attitudes and adverse events [19–26]. Few studies, however, have specifically examined changes in individual abortion provider performance with interventions encompassing extended post-training contacts with providers from support teams skilled in clinical and programmatic dimensions of abortion care in health facilities.

This paper reports findings from a multi-faceted intervention to improve individual abortion provider performance in health facilities in India, Nepal and Nigeria. These three countries were chosen because each country had Ipas-supported programs to improve abortion care for five or more years and demonstrated overall fidelity to the programmatic intervention model to train and provide post-training support to providers and health facilities. In addition, analyzing the results collectively across these countries was intended to identify factors associated with achieving provider performance that would be relevant to a range of country settings. While India, Nepal and Nigeria all have legal indications for abortion, provision of safe, legal abortion and treatment of complications from spontaneous and unsafely induced abortion vary widely based on country context [27].

Nigeria has the most restrictive law, only permitting abortion to save the life of the woman [27]. However, induced abortion is widely practiced by trained providers who broadly interpret the legal provisions, especially in the private sector, and by untrained providers and women themselves, which often require treatment for abortion complications. India and Nepal both have more liberal indications for abortion. While India has had a relatively liberal abortion law since 1971, which allows abortion on socioeconomic grounds, restrictions on provision of care by physicians have limited women’s access to safe abortion. The Medical Termination of Pregnancy Act was updated in 2002 to allow for provision of abortion with medications; however, efforts to amend the law to allow for provision of abortion by other cadres of providers are ongoing. The law in Nepal, liberalized in 2002, allows for abortion without restriction. Auxiliary nurse midwives (ANMs) can provide induced abortion with medications and treat abortion complications with manual vacuum aspiration, making services increasingly more available in rural areas.

The intervention under examination was implemented by health systems and facilities in the three countries in partnership with Ipas, a global, reproductive health technical assistance organization, and the Ipas Development Foundation for India. The objectives are to: 1) Describe the specific interventions implemented to enhance performance; 2) Identify those factors related to provider experience, facility environment and post-training support offered that are most likely to strengthen abortion provider performance; and 3) Develop recommendations to reinforce abortion provider performance and identify remaining gaps in our current knowledge base.

## Methods

### Study aim, design and sample

This study aimed to examine changes in individual abortion provider performance resulting from an intervention encompassing post-training support contacts to improve their clinical practice and resolve programmatic problems. The study design was a non-experimental, longitudinal cohort of providers who attended a clinical training on comprehensive abortion care and subsequently received at least one full year of post-training support between July 2011 and June 2016.

### Interventions to improve provider performance

The model for improving performance included selection of appropriate facilities and providers, preparation of facilities for service provision, training of providers, and offering follow-up clinical and programmatic support to providers, as much and as often as needed, to achieve performance levels. Table 1 summarizes the intervention components by country.

**Table 1:** Components of provider-improvement intervention, by level and country

Intervention Components	India	Nepal	Nigeria
Individual Provider Level			
Meets prerequisites for service provision	X	X	X
Competency-based training to improve knowledge, counseling and clinical skills	X	X	X
Cadre trained/length of training	MBBS physicians/5 days	Auxiliary nurse midwives/5 days Physicians/10 days	Physicians & midwives/5 days
Values Clarification and Attitude Transformation exercises	X	X	X
Clinical mentoring – coaching, individualized feedback, demonstration, problem-solving, case review, job aids	X	X	X
Motivation – encouragement and recognition of providers	X	X	X
Programmatic support contacts – Logistics, problem-solving, service data audit/review	X	X	X
Provider networking	Virtual, online website	In-person	In-person
Health Facility Level			
Improved infrastructure	X	X	X
Sustainable logistics for supplies/commodities	X	X	X
Performance/Quality improvement activities (some facilities)	X	X	X
Improved abortion case recordkeeping	X	X	X
Community Level			
Outreach, awareness-raising and referral for care	As needed	Community Health Volunteers	Lack of resources to employ

Facilities eligible for selection met one of the following criteria: 1) Facility was authorized to provide abortion care in national standards and guidelines; or 2) Facility could meet national standards by completing any required authorization, registration, or with modest investments in infrastructure and supplies. Additional factors considered in selection included facility staffing, data on any abortion care provided at the facility and the relative location of the facility to other service delivery points. Following selection, intervention facilities were upgraded to provide care to standards, ideally on an outpatient basis, with contraceptive services provided at the same location as abortion-related procedures [28]. Supplies were provided for service initiation with subsequent support focused on sustainable procurement by the public health system or by private providers.

Providers were chosen to be trained if they met the following clinical and legal prerequisites: 1) Knowledge of reproductive system and anatomy; 2) Ability to perform a bimanual exam; 3) Knowledge and skills in contraceptive counseling and provision for a general population; and 4) Authorization by country standards and guidelines to provide abortion care. Additionally, providers who self-reported a willingness to provide services were prioritized for training. Providers participated in Values Clarification and Attitude Transformation exercises [29] to ensure correct knowledge of their country’s abortion law, clarify personal attitudes about contraception and abortion and separate personal attitudes from professional obligations to provide care.

A significant component of the intervention was competency, performance-based training in comprehensive, woman-centered abortion care that included client counseling, uterine evacuation for induced abortion and/or PAC, provision of postabortion contraceptive methods, record-keeping and other content. Training curricula were based on adult-learning principles and participatory methods for classroom, simulated practice and supervised clinical practicum, adapted to the local context [30]. At the time of training, providers were judged clinically competent if abortion procedures and related care were performed correctly as determined by a clinical trainer utilizing a competency checklist. Providers self-assessed their confidence in performing procedures at completion of training.

Post-training support was designed to respond to providers’ individual needs, using approaches such as additional clinical mentoring, case review and problem-solving, motivation and encouragement, audit and feedback, supportive supervision, job aids and resolution of logistics issues that collectively may have great impact on performance [3, 8, 16]. A provider support team was designated for each trained provider. The composition of the provider support team was tailored to the context of the health system in each country, and typically included a clinical mentor, who provided clinical inputs, together with health system personnel and Ipas technical assistance staff who offered programmatic support. Facility and community level interventions were also implemented based on each country’s resources to raise community level awareness about unsafe abortion and inform and refer women for services.

Those providers not judged clinically competent were required to have an in-person clinical mentor visit within three weeks post-training to provide additional supervised clinical practice. All other providers were scheduled to receive a programmatic support contact, virtually or in-person, usually three to four weeks post-training. Provider support team members periodically contacted providers by telephone or in-person, as needed and for regularly-scheduled visits, to discuss progress and challenges, observe patient care and provide clinical and programmatic guidance based on performance gaps. Building on use of performance reports to positively influence provider behavior [31], summarized service data was shared with providers. Specific numeric performance levels were not communicated to individual providers to avoid the potential of coercive behavior toward clients to meet performance measures. Providers were given contact information for clinical mentors and programmatic support persons on their provider support team, whom they could contact at any time when they required assistance.

During visits to individual providers by support team members, support also included discussions with facility supervisors and other staff at the facility to address issues that affected individual provider performance. Some facilities also carried out performance quality improvement activities from which individual providers may have benefitted. Each country also developed a provider network based on their country context and resources, which included virtual and/or in-person exchanges and technical updates. Provider networks were intended to encourage peer support for service provision, share lessons learned and successful approaches to improving service provision and in some instances to recognize high performance or improved performance of providers.

### Data collection

Five data sources were used as part of routine monitoring of the intervention in each country. Baseline questionnaires were administered to providers prior to clinical training to assess their confidence, competence and experience providing abortion and contraceptive services. Similarly, baseline questionnaires were administered at the main facilities where the providers practiced by a combination of Ipas or Ipas Development Foundation staff and hired consultants. This information assessed health facilities’ preparedness to provide abortion and contraceptive services. Activity forms for all clinical trainings were also collected by programmatic staff, detailing the location, trainers, start and end dates, content covered and participants in attendance.

Information on post-training provider support was captured with a structured questionnaire called a provider progress report (PPR). PPRs were administered during exchanges with the providers by the provider support team members. The PPR collected information on providers’ self-reported provision of abortion and contraceptive services following training, clinical and programmatic issues they experienced, and clinical and programmatic support given to the providers. PPR forms were collected at every contact between a member of the support team and a trained provider, either through virtual contacts (short message service (SMS), phone, email) or in-person contacts.

At the initial training, providers were trained to record client-level data in facility logbooks on an ongoing basis. The contents of abortion logbooks varied by country, but certain categories were universal. These included diagnosis (induced abortion or PAC), technology used for uterine evacuation [manual or electric vacuum aspiration, MVA/EVA, dilation and evacuation (for second-trimester procedures), sharp curettage and medical abortion], pregnancy gestation, and client uptake of postabortion contraception by method. Trained providers were assigned a logbook identification code to record for each case they attended, allowing performance of each individual provider to be monitored. Data on pain management administration for surgical uterine evacuation methods such as MVA/EVA and sharp curettage were only collected in logbooks in Nigeria and Nepal. Providers in India were trained in pain management, but the abortion logbooks in government health facilities do not record this indicator. Logbook data were collected by project staff/consultants quarterly (Nepal, Nigeria) or every six months (India). No client names, numbers or other identifying information were collected. All data were entered into an online monitoring and evaluation database managed by the technical assistance organization.

### Performance measures

Four indicators were used to measure post-training performance. These indicators were:

1) Increase in service provision; 2) Consistent service provision; 3) Provision of high quality of care through use of WHO-recommended uterine evacuation technologies, management of pain and provision of postabortion contraception; and 4) Postabortion contraception method mix. All indicators were calculated beginning with the final day of the provider’s clinical training and their subsequent service provision as recorded in facility logbooks. Analysis was limited to the time period of 12-months post-training for each provider and calculated as a Didn’t Meet/Met (0 = No; 1 = Yes) variable to indicate if the provider achieved that particular level of performance. The following provides the rationale for use of these indicators, recommended levels and methods for collecting and analyzing the measures.

Increasing the number and proportion of clients out of all those needing a service is a common expectation of public health interventions. For purposes of this intervention, a 20% increase in the number of abortion procedures provided post-training was set as a performance level. However, in low-resource settings, it is difficult to estimate the level of service increase necessary to meet expected need for safe abortion services, given the absence of population-level abortion rates, poor reporting quality of abortion data and legal restrictions on abortion availability. The increase in service provision was met if the provider increased her/his monthly provision of abortion procedures by at least 20% from their first month of service provision post-training compared to the average of all subsequent months in the time period. If a provider was delayed in initiation of abortion services following training, we excluded the months of no services from the analysis. Providers who did not begin service provision within nine months post-training were excluded from this outcome, because at least three months of caseload data were required for calculation. Providers who did not provide any services during the first year post-training were included in the analysis and considered as not having met this performance measure.

Consistent service provision, in part, reflects provider ability to maintain skill level and to care for women, rather than unnecessarily referring or turning away clients. However, consistent service provision is also influenced by other factors beyond individual provider performance, such as seasonal variations in women’s care-seeking behavior. For purposes of this intervention, consistent service provision was defined as provision of at least three abortion services per quarter (average of one service per month) following the commencement of service provision post-training. Providers who did not begin service provision within six months post-training were excluded from this outcome, as two full quarters (six months) were required to calculate the measure. Providers who did not provide services entirely during the first year post-training were included in the analysis and considered not to have met the outcome.

Provision of high-quality abortion services encompasses three elements of care – the use of WHO- recommended [32] abortion technologies for uterine evacuation (MVA/EVA and medical abortion), use of pain management, and postabortion contraception provision at the time of care. While quality of abortion care has been defined in various ways [33], these three elements are important process indicators that contribute positively to safety and women’s experience of care. Outcome measures such as post-procedure complications, contraception continuation or subsequent pregnancy were not utilized due to cost and methodological difficulties in data collection. Providers were expected to use recommended technologies for 95% of first-trimester abortion patients/clients and to provide pain management for 95% of first-trimester procedures performed with surgical methods of evacuation (MVA/EVA).

Providers were anticipated to provide postabortion contraception to 60% of women they cared for, using the most effective (intrauterine device (IUD), implant or sterilization) and more effective (pills, injectables) methods. While providers were trained to provide condoms, and condoms were made available to women who selected them, this method was not included in the calculation of contraceptive provision. The 60% level of postabortion contraception provision by a provider is similar to levels recommended for facility performance for contraception [20, 34, 35]. Providers had to achieve all three elements at the recommended level in order to have met the quality performance indicator.

The final performance measure was the contraceptive method mix provided to clients. Adequate postabortion method mix was defined as provision of at least three different contraceptive methods, at least one of which was a long-acting reversible contraceptive (LARC) method or permanent method. LARC methods can help women more effectively prevent unplanned pregnancy [36]. With increasing focus on LARC methods, concerns have also been raised about the potential for coercion and abuse of clients [37]. Setting levels of contraceptive provision by individual providers as a performance measure is also challenging because the indicator relies on individual women’s circumstances, method preferences and choices that are not wholly related to the clinician’s ability to counsel and provide specific methods. For these reasons, the National Quality Forum’s [34] recent recommendations do not establish LARC provision levels, but recommend identification of low levels of LARC provision that indicate the potential for needed intervention. Similarly, for this analysis we did not utilize a recommended level of LARC provision by providers, but incorporated LARC into an indicator for recommended overall method mix.

Measurements of post-training follow-up support to providers were the primary independent variables. Although eight variables were originally examined from the PPRs, the analysis was narrowed to two variables: 1) Total number of in-person contacts documented, and 2) Total number of virtual (SMS, email, phone) documented contacts. Thirty-one additional independent variables were included in the analysis to examine provider and site characteristics for each provider in the sample. These included 18 variables from the baselines on sites where trained providers practiced, including site level (primary, secondary, tertiary) and if the site had an outreach program that included abortion information shared in the community. Thirteen provider variables were also examined, including the provider cadre (Ob/Gyn specialist, general practitioner, or midlevel practitioner such as a midwife or clinical officer), fiscal year of the provider’s training, prior experience performing abortion procedures, and if other providers trained in comprehensive abortion care were also practicing in the facility.

### Data analysis

Analysis was performed using Stata/SE 14.2 and produced the percentage of providers in the sample who met the specific performance level. Descriptive univariate analysis was conducted of all dependent and independent variables, by country and overall. Bivariate relationships were examined between all independent variables and the four dependent performance outcome variables by calculating unadjusted odds ratios, by country and overall. Finally, multivariate logistic regression was performed for each outcome, including all countries in the models.

Regression diagnostics were conducted to test for collinearity, during which several independent variables considered in the model were dropped. These included provision of abortion services at the provider’s facility at baseline, the site sector (public or private/NGO), and the number of weeks between the provider’s training and first post-training contact. An interaction term was added to the models which adjusted for providers from India who had logbook services from secondary, non-intervention sites, as these providers were more likely to have improved performance. Although the direction of this association is unclear, it suggests that providers who were performing services at secondary facilities were likely to be performing better in both facilities rather than providing lower quality services at their secondary facility.

All models also adjusted for country and were clustered on facility. Variance accounting for within-facility correlation of outcomes was adjusted for using the cluster option in Stata/SE 14.2. Services delivered at any one facility were expected to be correlated because of the presence of the same set of providers with the same set of site-level constraints. Significance levels were set at 0.05.

## Results

A total of 3471 providers were included in the sample, including 2671 providers in India, 542 in Nepal, and 258 in Nigeria. Training occurred over a four-year period during 2012–2015, although the program in Nepal trained a small number of providers in 2010 and 2011 [findings included only in Table 2]. Response rates for the questionnaires were high, with 99% of providers (3435) completing the provider questionnaire and 98% of facilities (2411) completing the site questionnaire. A total of 19,794 PPRs were collected during the providers’ first year post-training, with 98% of providers (3386) receiving at least one documented support contact during the period. Almost three-quarters of providers worked in primary level health facilities, although less than one-half of Nigerian providers did so. The providers in the sample practiced in 2461 facilities, 1944 in India, 325 in Nepal, and 192 in Nigeria.

**Table 2:** Characteristics of providers and post-training support provided, by country and overall

	India (*n* = 2671)		Nepal (*n* = 542)		Nigeria (*n* = 258)		Overall (*n* = 3471)	
	n	(%)	n	(%)	n	(%)	n	(%)
Provider Characteristics								
Provider type								
Ob/Gyn specialist	563	21%	43	8%	14	5%	620	18%
General practice physician	2108	79%	7	1%	142	55%	2257	65%
Midlevel	0	0%	492	91%	102	40%	594	17%
Fiscal year trained								
2010	0	0%	8	1%	0	0%	8	<1%
2011	0	0%	7	1%	0	0%	7	<1%
2012	691	26%	130	24%	157	61%	978	28%
2013	752	28%	141	26%	35	14%	928	27%
2014	607	23%	115	21%	0	0%	722	21%
2015	621	23%	141	26%	66	26%	828	24%
Any prior uterine evacuation experience								
Yes	677	25%	196	38%	208	86%	1081	31%
No	1994	75%	325	62%	34	14%	2353	69%
Missing	0		21		16		37	
Number of abortion providers currently at site, including index provider								
1	2088	78%	209	39%	120	47%	2417	70%
2	337	13%	151	28%	84	33%	572	16%
3+	246	9%	182	34%	54	21%	482	14%
Site Characteristics								
Site level								
Primary	1927	72%	462	85%	120	47%	2509	72%
Secondary	622	23%	65	12%	132	51%	819	24%
Tertiary	122	5%	15	3%	6	2%	143	4%
Community outreach with abortion information								
Yes	722	27%	262	56%	42	18%	1026	31%
No	1916	73%	210	44%	192	82%	2318	69%
Missing	33		70		24		127	
Post-training support received during 12 months post-training								
Total number of post-training contacts with provider								
Mean	5.9		2.5		9.9		5.7	
(SD)	2.4		1.8		4.9		3.1	
Total number of in-person, post-training contacts with provider								
Mean	3.3		2.0		7.5		3.4	
(SD)	1.6		1.5		3.8		2.3	
Any program support contacts								
Yes	2669	100%	289	53%	18	7%	2976	86%
No	2	0%	253	47%	240	93%	495	14%
Any clinical mentoring contacts								
Yes	701	26%	168	31%	17	7%	886	26%
No	1970	74%	374	69%	241	93%	2585	74%

Almost two-thirds of providers were general practice physicians, reflecting high percentages of this cadre in India and Nigeria (79% and 55%, respectively) [Table 2]. Overall, less than one-third of providers had performed uterine evacuations for induced abortion or treatment of incomplete abortion prior to the training, although a large majority (86%) of Nigerian providers were experienced [Table 2]. At training, providers reported if their facility currently offered outreach efforts to the community that included abortion information. Less than one-third of providers indicated these activities were part of their facility’s activities [Table 2].

Individuals from the provider support teams, and in some cases Ministry of Health supervisors, made an average of 5.7 post-training contacts with each provider during the 12-month period, although the average by country varied widely. Nigerian providers received almost 10 contacts, while Nepali providers had 2.5. In Nepal, most of the contacts were in-person (2.0), with a much lower average in India (3.3) and Nigeria (7.5) [Table 2]. Most contacts (86%) focused on programmatic, non-clinical issues such as resolving gaps in commodities’ supplies, difficulties in the management of abortion services and enhancing the organization and efficiency of abortion services. Just 26% of the contacts involved clinical mentoring such as improving providers’ surgical techniques [Table 2].

Outcome measures for the 3471 providers during the 12 months were based on analysis of 166,067 cases, including 122,350 in India, 12,177 in Nepal and 31,540 in Nigeria. Achievement of the four outcome measures did not vary markedly from one country to another, with a few exceptions [Table 3]. A lower percentage of Nigerian providers achieved the quality of care measure, compared to those in India and Nepal. Providers in Nepal and Nigeria had more difficulty reaching the postabortion contraceptive uptake measure that those in India, while Indian and Nepali providers had lower achievement of the contraceptive method mix indicator compared to Nigerian providers.

**Table 3:** Provider performance outcomes by 12 months post-training, by country and overall

	India (*N* = 2671)		Nepal (*N* = 542)		Nigeria (*N* = 258)		Overall (*N* = 3471)	
	n	(%)	n	(%)	n	(%)	n	(%)
Provider Performance Outcomes by 12 Months Post-Training								
Increase in abortion service provision								
Yes	665	25%	89	17%	64	26%	818	24%
No	1985	75%	449	83%	186	74%	2620	76%
Missing	21		4		8		33	
Consistency in abortion service provision								
Yes	1614	63%	287	56%	152	64%	2053	62%
No	966	37%	225	44%	86	36%	1277	38%
Missing	91		30		20		141	
Provision of high-quality abortion services								
Yes	1305	49%	217	40%	79	31%	1601	46%
No	1366	51%	325	60%	179	69%	1870	54%
95% Provision of pain management medication or referral								
Yes	N/A^a^		490	90%	216	84%	706	88%
No			52	10%	42	16%	94	12%
95% Use of appropriate evacuation technology								
Yes	2186	82%	489	90%	222	86%	2897	83%
No	485	18%	53	10%	36	14%	574	17%
At least 60% contraceptive uptake at time of service (excluding condoms)								
Yes	1438	54%	220	41%	89	34%	1747	50%
No	1233	46%	322	59%	169	66%	1724	50%
Provision of adequate contraceptive method mix at time of service (including condoms)								
Yes	1258	47%	195	36%	152	59%	1605	46%
No	1413	53%	347	64%	106	41%	1866	54%

^a^Pain management data not collected in Indian facility logbooks

Increased service provision was the most difficult outcome for all providers to achieve with less than one-quarter (24%) doing so [Table 3]. Almost two-thirds (62%) of providers reached the consistency outcome. Forty-six percent of providers achieved the quality outcome. Provision of pain management and use of appropriate evacuation technology were high across the three countries (88% and 83%, respectively), while overall achievement of the postabortion contraceptive component was relatively low (50% of providers) [Table 3].

The adjusted analysis showed that providers working at secondary- and tertiary-level facilities were more likely to reach the increased service provision outcome than those at primary sites, but the differences were not statistically significant (secondary-level OR:1.2, CI:0.95–1.51; tertiary level-OR:1.78, CI:0.99–3.19) [Table 4]. Providers working at facilities that offered community outreach incorporating abortion information had significantly higher odds of reaching the increased number of services outcome than those without outreach (OR:1.26, CI:1.03–1.55) [Table 4]. Other factors in the model that were significantly associated with increased services were those providers trained in 2013 (OR:1.56, CI:1.24–1.96) and those receiving a higher number of in-person, post-training contacts (OR:1.17; CI:1.11–1.22) [Table 4].

**Table 4:** Unadjusted and adjusted odds ratios for abortion provider performance: Increase in number of abortions provided

	Bivariate associations (*n* = 3438)						Adjusted odds ratios (*n* = 3293)	
	Increased = No		Increased = Yes		OR	95% CI^1^	AOR^a^	95% CI^1^
	n	(%)	n	(%)				
Site Characteristics								
Site level								
Primary	1932	78%	552	22%	Ref		Ref	
Secondary	594	73%	218	27%	1.28	(1.07, 1.54)**	1.20	(0.95, 1.51)
Tertiary	94	66%	48	34%	1.78	(1.25, 2.56)**	1.78	(0.99, 3.19)
Community outreach with abortion information								
Yes	763	75%	256	25%	1.10	(0.92, 1.90)	1.26	(1.03, 1.55)*
No	1756	77%	538	23%	Ref		Ref	
Provider Characteristics								
Fiscal year trained								
FY 2012	747	77%	220	23%	Ref		Ref	
FY 2013	650	71%	271	29%	1.44	(1.17, 1.77)**	1.56	(1.24, 1.96)***
FY 2014	555	77%	162	23%	1.01	(0.80, 1.27)	0.97	(0.75, 1.26)
FY 2015	654	80%	165	20%	0.87	(0.70, 1.10)	0.81	(0.63, 1.04)
Any prior uterine evacuation experience								
Yes	803	75%	273	25%	1.13	(0.95, 1.33)	0.95	(0.77, 1.17)
No	1788	77%	540	23%	Ref		Ref	
Any other Ipas-trained providers at site								
Yes	803	77%	239	23%	0.93	(0.79, 1.11)	0.95	(0.76, 1.19)
No	1817	76%	579	24%	Ref		Ref	
Provider Support								
Total in-person, post-training contacts (Mean, SD)	(3.3, 2.2)		(3.8, 2.4)		1.11	(1.07, 1.15)***	1.17	(1.11, 1.22)***
Total non-in-person, post-training contacts (Mean, SD)	(2.3, 2.0)		(2.3, 1.8)		1.01	(0.97, 1.05)	0.95	(0.91, 1.00)

^a^Adjusted odds ratios control for country variations, an interaction term identifying Indian providers who performed UE at secondary sites, and are clustered on facilities

^1^Significant at the following levels: *p < 0.05; **p < 0.01; ***p < 0.001

In the adjusted model, providers had significantly higher odds of offering consistent services if they worked at a site with community outreach on abortion (OR:1.33, CI:1.10–1.60), were trained in 2013 (OR:2.02; CI:1.63–2.49), reported prior uterine evacuation experience (OR:1.68, CI:1.38–2.04) or received a higher number of in-person, post-training contacts (OR:1.28; CI:1.22–1.35) [Table 5].

**Table 5:** Unadjusted and adjusted odds ratios for abortion provider performance: Consistent number of abortions provided

	Bivariate associations (*n* = 3330)						Adjusted odds ratios (*n* = 3194)	
	Consistent = No		Consistent = Yes		OR	95% CI^1^	AOR^a^	95% CI^1^
Site Characteristics	n	(%)	n	(%)				
Site level								
Primary	974	41%	1425	59%	Ref		Ref	
Secondary	258	33%	533	67%	1.41	(1.19, 1.67)***	1.25	(0.99, 1.58)
Tertiary	45	32%	95	68%	1.44	(1.00, 2.08)*	1.02	(0.48, 2.14)
Community outreach with abortion information								
Yes	361	36%	633	64%	1.13	(0.97, 1.32)	1.33	(1.10, 1.60)**
No	871	39%	1348	61%	Ref		Ref	
Provider Characteristics								
Fiscal year trained								
FY 2012	400	43%	530	57%	Ref		Ref	
FY 2013	271	30%	627	70%	1.80	(1.49, 2.18)***	2.02	(1.63, 2.49)***
FY 2014	262	38%	434	62%	1.29	(1.06, 1.58)*	1.25	(0.99, 1.57)
FY 2015	331	42%	462	58%	1.09	(0.90, 1.32)	0.99	(0.80, 1.23)
Any prior uterine evacuation experience								
Yes	305	29%	746	71%	1.81	(1.54, 2.11)***	1.68	(1.38, 2.04)***
No	954	42%	1292	58%	Ref		Ref	
Any other Ipas-trained providers at site								
Yes	399	40%	610	60%	0.93	(0.80, 1.08)	0.85	(0.68, 1.06)
No	878	38%	1443	62%	Ref		Ref	
Provider Support								
Total in-person, post-training contacts (Mean, SD)	(2.9, 2.0)		(3.7, 2.3)		1.20	(1.16, 1.25)***	1.28	(1.22, 1.35)***
Total non-in-person, post-training contacts (Mean, SD)	(2.3, 2.0)		(2.3, 1.9)		0.99	(0.96, 1.03)	0.93	(0.89, 0.98)**

^a^Adjusted odds ratios control for country variations, an interaction term identifying Indian providers who performed UE at secondary sites, and are clustered on facilities

^1^Significant at the following levels: *p < 0.05; **p < 0.01; ***p < 0.001

Providers were significantly more likely to achieve the quality of care outcome in the adjusted model if their sites offered abortion-related community outreach (OR:1.46, CI:1.21–1.75), they were trained in 2013 (OR:1.30, CI:1.05–1.61), or other Ipas-trained providers also worked at their facility (OR:1.56, CI:1.25–1.95) [Table 6].

**Table 6:** Unadjusted and adjusted odds ratios for abortion provider performance: Provision of high-quality services

	Bivariate associations (*n* = 3471)						Adjusted odds ratios (*n* = 3322)	
	High Quality = No		High Quality = Yes		OR	95% CI^1^	AOR^a^	95% CI^1^
Site Characteristics	n	(%)	n	(%)				
Site level								
Primary	1298	52%	1211	48%	Ref		Ref	
Secondary	467	57%	352	43%	0.81	(0.69, 0.95)**	0.78	(0.62, 0.99)*
Tertiary	105	73%	38	27%	0.39	(0.27, 0.57)***	0.29	(0.10, 0.87)*
Community outreach with abortion information								
Yes	486	47%	540	53%	1.40	(1.21, 1.63)***	1.46	(1.21, 1.75)***
No	1294	56%	1024	44%	Ref		Ref	
Provider Characteristics								
Fiscal year trained								
FY 2012	563	58%	415	42%	Ref		Ref	
FY 2013	470	51%	458	49%	1.35	(1.13, 1.62)**	1.30	(1.05, 1.61)*
FY 2014	364	50%	358	50%	1.36	(1.12, 1.65)**	1.17	(0.94, 1.47)
FY 2015	459	55%	369	45%	1.12	(0.93, 1.34)	1.01	(0.82, 1.26)
Any prior uterine evacuation experience								
Yes	669	62%	412	38%	0.61	(0.53, 0.71)***	0.74	(0.61, 0.90)**
No	1172	50%	1181	50%	Ref		Ref	
Any other Ipas-trained providers at site								
Yes	548	52%	506	48%	1.11	(0.96, 1.29)	1.56	(1.25, 1.95)***
No	1322	55%	1095	45%	Ref		Ref	
Provider Support								
Total in-person, post-training contacts (Mean, SD)	(3.5, 2.4)		(3.3, 2.1)		0.97	(0.94, 1.00)*	1.00	(0.96, 1.05)
Total non-in-person, post-training contacts (Mean, SD)	(2.3, 2.0)		(2.3, 1.9)		1.01	(0.97, 1.04)	0.95	(0.91, 1.00)*

^a^Adjusted odds ratios control for country variations, an interaction term identifying Indian providers who performed UE at secondary sites, and are clustered on facilities

^1^Significant at the following levels: *p < 0.05; **p < 0.01; ***p < 0.001

Table 7 reports the bivariate and adjusted analysis for providers who provided an adequate contraceptive method mix to their abortion clients. Factors significantly associated with adequate method mix in the model were practicing at a facility offering community outreach with abortion information (OR:1.46, CI:1.22–1.76), being trained in 2013 (OR:1.30, CI:1.06–1.60), having previous uterine evacuation experience at time of the intervention training (OR:2.24, CI:1.86–2.70), working at facilities with other providers trained in the intervention (1.31, CI:1.06–1.62), or receiving a higher number of in-person contacts following training (OR:1.11, CI:1.06–1.16) [Table 7].

**Table 7:** Unadjusted and adjusted odds ratios for abortion provider performance: Provision of adequate contraceptive method mix

	Bivariate associations (*n* = 3471)						Adjusted odds ratios (*n* = 3322)	
	Adequate FP method mix = No		Adequate FP method mix = Yes		OR	95% CI^1^	AOR^a^	95% CI^1^
Site Characteristics	n	(%)	n	(%)				
Site level								
Primary	1446	58%	1063	42%	Ref		Ref	
Secondary	362	44%	457	56%	1.72	(1.47, 2.01)***	1.15	(0.91, 1.43)
Tertiary	58	41%	85	59%	1.99	(1.41, 2.81)***	1.16	(0.60, 2.24)
Community outreach with abortion information								
Yes	512	50%	514	50%	1.26	(1.09, 1.46)	1.46	(1.22, 1.76)***
No	1292	56%	1026	44%	Ref		Ref	
Provider Characteristics								
Fiscal year trained								
FY 2012	534	55%	444	45%	Ref		Ref	
FY 2013	454	49%	474	51%	1.29	(1.08, 1.54)**	1.30	(1.06, 1.60)*
FY 2014	399	55%	323	45%	1.00	(0.83, 1.21)	0.93	(0.75, 1.17)
FY 2015	464	56%	364	44%	0.97	(0.81, 1.17)	0.85	(0.69, 1.06)
Any prior uterine evacuation experience								
Yes	422	39%	659	61%	2.37	(2.04, 2.75)***	2.24	(1.86, 2.70)***
No	1419	60%	934	40%	Ref		Ref	
Any other Ipas-trained providers at site								
Yes	525	50%	529	50%	1.26	(1.09, 1.45)**	1.31	(1.06, 1.62)*
No	1341	55%	1076	45%	Ref		Ref	
Provider Support								
Total in-person, post-training contacts (Mean, SD)	(3.1, 2.0)		(3.7, 2.4)		1.13	(1.10, 1.17)***	1.11	(1.06, 1.16)***
Total non-in-person, post-training contacts (Mean, SD)	(2.3, 2.0)		(2.3, 1.9)		1.00	(0.77, 0.95)**	0.97	(0.93, 1.01)

^a^Adjusted odds ratios control for country variations, an interaction term identifying Indian providers who performed UE at secondary sites, and are clustered on facilities

^1^Significant at the following levels: *p < 0.05; **p < 0.01; ***p < 0.001

Additional analysis of each performance outcome excluding India’s results, recognizing its large contribution to the provider sample, revealed that removing India from the model caused the results for 2013 to lose significance for all four of the outcomes (data not shown). Table 8 summarizes the factors that significantly affected providers’ achievement of each of the four performance measures.

**Table 8:** Variables associated with elements of abortion provider performance in adjusted logistic regression models

	Abortion Provider Performance Outcomes by 12-months post-training			
	Increase in number of abortions provided	Consistent number of abortions provided	Provision of high quality abortion services	Provision of adequate contraceptive method mix
Site Characteristics				
Site level^a^			X	
Community outreach with abortion information	X	X	X	X
Provider Characteristics				
Any prior uterine evacuation experience		X		X
Any other Ipas-trained providers at site			X	X
Provider Support				
Total in-person, post-training contacts (Mean, SD)	X	X		X

X = Adjusted OR significant at the *p* < 0.05 level

^a^Primary level sites were positively associated with achievement of high quality performance when compared to secondary and tertiary level sites

## Discussion

The intervention described in this paper incorporated components at the individual provider, health facility and community levels, reflecting evidence that multiple interventions to improve health worker performance are more effective than single interventions [2]. The training curriculum encompassed diverse methodologies shown to be particularly effective for health workers, such as use of case studies, clinical simulations, skills practice and feedback to learners [13]. Recognizing that health workers require an appropriate environment in which to practice, the intervention also included improvements to health facilities [10]. Community education has been shown to be effective when combined with other interventions focused on health workers [2]; two of the three country interventions also included community outreach and referrals.

Post-training follow-up and support contacts of providers who had completed an abortion care training course was a key component of the intervention. A number of studies have examined the effectiveness of various types of follow-up of health workers, including supportive supervision, coaching, mentoring and others. While Rowe et al. [2] found that supervision plus audit with feedback had moderate to large effects on performance, other reviews report inconsistencies in definitions of supervision as well as uncertain or limited effects on quality of care and clinical outcomes [8, 10, 11].

Intervention studies of health worker performance have evaluated a variety of measures, including job satisfaction, clinical knowledge and skills, and practice [2, 3]. During the abortion care training and in follow-up contacts, the support teams in this study’s intervention assessed providers’ knowledge and skills. The outcomes reported in this paper (increase in and consistency of service provision, quality of care and postabortion contraceptive method mix) are measures of provider practice. All outcomes reflect actual care given to clients by providers who had been trained and followed-up; several measures have not been defined, assessed and reported in the abortion care literature to date. Although included in many studies of health worker performance, quality of care is often not a well-defined outcome [13]. This analysis aimed to address this gap through use of a multi-dimensional, quantitative measure used in prior studies and available in facility logbook records of care provided [20].

Almost two-thirds of providers across the three countries achieved the consistency performance measure, and less than one-half met the quality of care and adequate contraceptive method mix measures. Most providers met two of the three quality sub-measures (use of WHO-recommended abortion technologies and pain management) while they experienced more difficulties achieving the postabortion contraceptive uptake indicator. Increase in the number of abortion services offered was the most challenging measure to achieve.

The factors significantly associated with achievement of all four measures were the availability of community outreach with abortion information that was offered by the providers’ facilities, and the year in which the provider was trained with 2013 showing the highest achievement of most indicators. While just under one-third of providers reported that their sites offered community outreach, this strong performance achievement underscores the potential for reaching more women with high-quality abortion care when communities are made aware of the service. This is particularly important in settings where abortion care is a new service offered. Most of the providers in our sample worked at primary-level sites where abortion care was not previously available; in Nepal and India, most of those trained were first-time abortion providers. These results reinforce the previously cited finding that community education combined with health worker interventions have a moderate effect on provider performance [2]. In a review of supportive supervision studies in sub-Saharan Africa, Bailey et al. [10] report that engaging community and local leaders enhances shared responsibility for problems and their solutions in primary health facilities.

Overall findings show statistically significant, improved performance in 2013, the second year of provider training, compared to the first year in 2012. However, removing India from the analysis caused the results for 2013 to lose significance for all four outcomes. 2012 was an intense year of learning, problem-solving and making adjustments in a new intervention for each country. For India especially, the intervention was implemented in geographically-dispersed states, with in-person follow-up of almost 700 providers working in remote, rural primary centers. The statistically significant performance improvements in the second year for India may reflect the experience gained by the provider support teams and additional efficiencies in implementation and data gathering. By the last year of training in 2015, the overall outcomes for the three countries combined were similar to those from 2012, although significant differences for individual outcomes in some countries remained. Overall similar findings for 2012 and 2015 may reflect the cumulative demands on the time and efforts of the support teams that were following-up providers trained in the three previous years, most of whom received support longer than the 12 month-period reported for our sample, as well as contacting, visiting and supporting the new providers trained in 2015.

Meeting the quality of care measure was significantly associated with almost all of the site, provider and post-training support characteristics. Achievement of high quality care, especially use of appropriate technologies for procedures and use of pain management, is more amenable to providers’ individual actions than other outcomes such as an increased number of women seeking services which are less under their control. Providers at secondary and tertiary-level facilities were less likely to meet the quality measure than those working in primary sites. Variations in quality improvements among different levels of health facilities have been found in other studies [14, 24]. In a postabortion contraceptive study in health facilities in eight countries, Benson et al. [24] noted higher uptake among clients cared for in primary level sites compared to those seen in hospitals.

As previously noted, many primary-level providers were new to abortion services, had recently been trained on abortion care quality and were putting their newly-learned skills into practice. Those providers with prior abortion practice experience were significantly less likely to meet the quality measure, underscoring the challenges of modifying long-standing clinical practice, even with a multi-faceted intervention conducted over time. Newly-trained providers were more likely to achieve the quality measure if they worked in a facility with coworkers who had previously been trained in comprehensive abortion care, perhaps reflecting the reinforcing nature of peer support in the workplace, especially of an often-stigmatized service. The number of post-training contacts had little effect on providers’ ability to meet the quality measure. Similarly, a recent review of interventions involving supportive supervision of primary care health workers concluded that while supervision is beneficial to performance, no clear guidance has emerged on the optimal frequency or timing of visits [11].

In contrast to the quality of care measure, achievement of the indicators to increase the number of women served and to provide consistent care are influenced by factors external to the provider, such as women’s choices of where to seek care, the availability of nearby facilities offering abortion and low caseloads in primary-level facilities. However, trained providers can affect contraceptive uptake as well as method mix by appropriate counseling on a range of methods. Multiple studies to evaluate supportive supervision demonstrate its highest impact on counseling and communications techniques [10]. Yet, these contraceptive-related outcomes are also influenced by the consistent availability of short- and long-acting commodities in the facility, women’s preferences for specific methods and other factors.

In-person post-training contacts were significantly associated with achievement of three of the four outcome measures. On average, the support teams visited the providers slightly fewer than four times during the 12-month period. These findings are consistent with other studies that found improved outcomes with health worker supervision, audit and feedback, components that were part of the post-training support offered to the providers in the sample [2, 6, 14]. Vasan et al. [11] report on the performance and outcome benefits of clinical mentoring of health workers by more experienced practitioners, usually involving observation of cases and individualized feedback, although the authors highlight the need for additional research on this approach.

It is important to bear in mind the difficulty of isolating the effect of post-training contacts from other components of the multi-faceted intervention, including provider training, strengthened logistics processes for abortion and contraceptive commodities and improved management support of the facility’s abortion service. Furthermore, individual health worker performance cannot be removed from the context of the health facility in which they practice. Numerous health system building blocks must be in place to permit providers to perform at the highest level of which they are capable, especially of a new or long-neglected, stigmatized service such as abortion care [10]. The impact of interventions that focus only on health worker performance may be lessened if their work environment is not also addressed.

This study had a number of advantages. The intervention was multi-faceted, with core components that were implemented at the individual provider, facility and community levels across the three countries. Most components were consistent with the current evidence base on health worker performance, and expected outcomes were clearly defined. Many studies on health worker performance are conducted in one country setting, while this analysis benefitted from the diversity of the legal, political, geographic and cultural environments, health systems and health care providers and communities in Nepal, India and Nigeria. While the abortion care literature is growing, few studies exist on which interventions are most effective in enhancing the performance of abortion care providers. This study should contribute to the overall field of health worker performance.

The intervention design had several limitations. Since resource constraints did not permit inclusion of a comparison group of providers who did not receive post-training support, this study is only able to determine associations between support and performance outcomes. Original record-keeping systems for abortion care were weak or non-existent in many facilities which precluded pre-intervention measures. In addition, it was difficult to systematically track the types of inputs given to providers in response to the problems they were encountering, and the authors were unable to draw conclusions about which specific intervention components were significantly associated with improved performance.

It is recommended that future research be designed to incorporate comparison groups of providers, ideally randomly selected, and assessment of different intervention components, including post-training support contacts, to determine those that produce the largest performance effects. In addition, the performance outcomes were set at a relatively high level, especially the achievement of an increased number of abortion clients. As noted, this measure was especially challenging for providers working in primary-level facilities with new abortion services and low caseloads. It could be useful to assess different and/or lower performance measures, for example, tailored to the facility level where the provider works. The current literature on abortion quality indicators should inform such measures [33, 35]. Finally, it is recommended that health systems consider the incorporation of individualized post-training support to providers of abortion, contraceptive and other reproductive health care to strengthen routine facility supervision.

## Conclusions

Evidence-based investments to improve health worker performance are essential to building the confidence and competence of individual providers and improving the health systems where they practice. These findings from a sizeable, diverse group of providers and environments indicate the potential benefit of individualized, post-training support to health workers as part of comprehensive interventions to strengthen the availability and quality of abortion care. Safe abortion services and postabortion care, offered by well-trained and supported providers, are fundamental to accelerated progress in women’s reproductive health and well-being.

## Abbreviations

EVA: Electric vacuum aspiration
IUD: Intrauterine device
LARC: Long-acting reversible contraceptive
LMICs: Low- and middle-income countries
MVA: Manual vacuum aspiration
NGO: Non-governmental organizational
PAC: Postabortion care
PHC: Primary health care
PPR: Provider progress report
SMS: Short message service
WHO: World Health Organization
